# Genome wide analysis of the transition to pathogenic lifestyles in Magnaporthales fungi

**DOI:** 10.1038/s41598-018-24301-6

**Published:** 2018-04-12

**Authors:** Ning Zhang, Guohong Cai, Dana C. Price, Jo Anne Crouch, Pierre Gladieux, Bradley Hillman, Chang Hyun Khang, Marc-Henri LeBrun, Yong-Hwan Lee, Jing Luo, Huan Qiu, Daniel Veltri, Jennifer H. Wisecaver, Jie Zhu, Debashish Bhattacharya

**Affiliations:** 1 Department of Plant Biology, Rutgers University, New Brunswick, New Jersey 08901 USA; 2 Department of Biochemistry and Microbiology, Rutgers University, New Brunswick, New Jersey 08901 USA; 3 United States Department of Agriculture, Agricultural Research Service; and Department of Botany and Plant Pathology, Purdue University, West Lafayette, Indiana, 47907 USA; 4 Department of Plant Biology, Center for Vector Biology, Rutgers University, New Brunswick, New Jersey 08901 USA; 5 Mycology and Nematology Genetic Diversity and Biology Laboratory, United States Department of Agriculture, Beltsville, Maryland 20705 USA; 6 BGPI, Univ Montpellier, INRA, CIRAD, Montpellier SupAgro, F-34398 Montpellier, France; 7 Department of Plant Biology, University of Georgia, Athens, GA 30602 USA; 8 National Institute of Agricultural Research, Avenue Lucien Brétignières, BP 01, 78850 Thiverval-Grignon, France; 9 Department of Agricultural Biotechnology, Center for Fungal Genetic Resources, Plant Genomics and Breeding Institute, and Research Institute of Agriculture and Life Sciences, Seoul National University, Seoul, 08826 Republic of Korea; 10 Department of Ecology, Evolution and Natural Resources, Rutgers University, New Brunswick, New Jersey 08901 USA; 11 Bioinformatics and Computational Biosciences Branch, Office of Cyber Infrastructure and Computational Biology, National Institute of Allergy and Infectious Diseases, NIH, Rockville, MD USA; 12 Department of Biochemistry, Purdue University, West Lafayette, Indiana, USA

## Abstract

The rice blast fungus *Pyricularia oryzae* (syn. *Magnaporthe oryzae*, *Magnaporthe grisea*), a member of the order Magnaporthales in the class Sordariomycetes, is an important plant pathogen and a model species for studying pathogen infection and plant-fungal interaction. In this study, we generated genome sequence data from five additional Magnaporthales fungi including non-pathogenic species, and performed comparative genome analysis of a total of 13 fungal species in the class Sordariomycetes to understand the evolutionary history of the Magnaporthales and of fungal pathogenesis. Our results suggest that the Magnaporthales diverged ca. 31 millon years ago from other Sordariomycetes, with the phytopathogenic blast clade diverging ca. 21 million years ago. Little evidence of inter-phylum horizontal gene transfer (HGT) was detected in Magnaporthales. In contrast, many genes underwent positive selection in this order and the majority of these sequences are clade-specific. The blast clade genomes contain more secretome and avirulence effector genes, which likely play key roles in the interaction between *Pyricularia* species and their plant hosts. Finally, analysis of transposable elements (TE) showed differing proportions of TE classes among Magnaporthales genomes, suggesting that species-specific patterns may hold clues to the history of host/environmental adaptation in these fungi.

## Introduction

The order Magnaporthales in the fungal class Sordariomycetes (Ascomycota) contains economically important pathogens of cereals and grasses, such as the rice blast fungus *Pyricularia oryzae* (syn. *Magnaporthe oryzae, Magnaporthe grisea*), the take-all pathogen of cereals *Gaeumannomyces graminis*, and the summer patch pathogen of turfgrasses *Magnaporthiopsis poae*. Over 200 species of Magnaporthales have been described, of which about 50% are pathogens of domesticated and wild monocotyledons.

The best studied species in Magnaporthales is the rice blast fungus, which was ranked number one on the “Top 10 fungal plant pathogens” list based on scientific and economic importance in a survey of 495 votes from the international plant mycology community^1^. Rice blast is the most destructive disease of rice, which serves as a staple food source for about one-half of the world’s population^2,3^. This pathogen also infects turfgrasses, causing gray leaf spot of perennial ryegrass and tall fescue. This multihost pathogen represents a significant threat to agriculture, as exemplified by recent emergence of wheat blast^4,5^.

In science, the rice blast fungus is a paradigm for understanding pathogen infection and numerous studies have been performed using this model system. The genome of this fungus was sequenced and released over a decade ago, and genome analysis revealed that it possesses a large set of secreted proteins, an expanded family of G-protein coupled receptors, and ~10% of repetitive elements^6,7^. The genomes of several other phytopathogenic species in the Magnaporthales have thereafter been sequenced^8^. However, the non-pathogen relatives remain neglected. The paucity of studies and the lack of sequence data from non-model species in Magnaporthales results in a poor understanding of the phylogeny and an unstable taxonomy of Magnaporthales species. Furthermore, genome data from non-pathogen lineages are necessary to provide a more robust comparative genomic framework to help place the rice blast fungus in an evolutionary context and to understand the evolution of pathogenesis and other characters of these important organisms.

To this end, we recently sequenced the genomes of five species and generated transcriptomes from 21 species of Magnaporthales, including saprobes and plant pathogens^9^. Phylogenomic analysis based on these and previously published genome data supported the existence of three major clades that correspond to their pathological characters: species in the “wood” clade are saprobes on submerged wood; species in the “blast” clade produce appressoria and infect leaf tissue of host plants; and those in the “root” clade generally produce hyphopodia and infect plant roots.

Here, we used comparative methods to analyze data from 13 fungal species in the class Sordariomycetes, including nine species in the Magnaporthales, two *Colletotrichum* species and two *Fusarium* species. The nine Magnaporthales species include two saprotrophic non-pathogens (*Ophioceras dolichostomum* and *Pseudohalonectria lignicola*) in the wood clade and seven cereal or grass pathogens (*Gaeumannomyces graminis, Magnaporthiopsis poae*, *M. incrustans*, *M. rhizophila, Nakataea oryzae* [syn. *Magnaporthe salvinii*]*, Pyricularia oryzae* and *P. grisea*) in the blast and root clades. The *Colletotrichum* and *Fusarium* species are also pathogens of plants. The over-arching goal of our study was to develop a comprehensive framework for understanding the evolutionary history of the Magnaporthales and of fungal pathogenesis, by comparing genomic data from plant pathogens and their non-pathogenic relatives. Specifically, we focused on the timeline for Magnaporthales diversification, the extent of horizontal gene transfer (HGT) in this lineage, evidence for positive selection in Magnaporthales genes as a result of environmental adaptation, the nature of the secretome and effectors, and the distribution of transposable elements (TEs) in these genomes.

## Results and Discussion

### Genome sequencing, assembly, and annotation

For the five Magnaporthales species sequenced for this project (i.e., *M. incrustans, M. rhizophila, N. oryzae, O. dolichostomum*, and *P. lignicola*), the raw sequence reads ranged from 10.2 to 29.6 million read pairs per species for genome data, and 16.4 to 32.6 million read pairs per species for transcriptome data (Table S1). The genome assemblies had a scaffold N50 values that ranged from 62 kbp (179 scaffolds, *N. oryzae*) to 252 kbp (52 scaffolds, *M. rhizophila*), and the contig N50 ranged from 30 kbp (344 contigs, *N. oryzae*) to 60 kbp (205 contigs, *M. incrustans*). There were substantial differences in the assembled genome sizes. *Nakataea oryzae* had the smallest genome (34.9 Mbp), whereas *O. dolichostomum* had the largest (43.0 Mbp). The GC-ratios of these genomes ranged from 54–58%. The smallest number of genes (12,077 genes) was predicted in the smallest genome, *N. oryzae*, whereas the largest number of genes (12,933 genes) was predicted in *M. incrustans*, which had a genome size of 39.3 Mbp. In the largest genome (*O. dolichostomum*), 12,519 genes were predicted (Table S2). *Magnaporthiopsis incrustans* had the largest contig N50, perhaps explaining the larger number of genes predicted from this genome.

To assess the gene inventories in the genome assemblies, CEGMA analyses were done to identify 248 low-copy, core eukaryotic genes. Complete copies of 235 (95%) to 237 (96%) genes were found (Table S3). If partial genes were counted, 97% to 98% of the core genes were found in the genomes, suggesting that the assemblies captured most of the genes in the target taxa. The missing genes were not randomly distributed; i.e., eight genes were missing in two or more genomes (KOG0062, KOG0261, KOG0434, KOG0969, KOG1123, KOG1185, KOG2311, KOG3232). Two genes, KOG1185 (thiamine pyrophosphate-requiring enzyme) and KOG2311 (NAD/FAD-utilizing protein possibly involved in translation) were missing in all five genome assemblies, indicating that these genes either do not exist in Magnaporthales or less likely, all reside in difficult-to-assemble DNA regions.

To facilitate dissemination of the Magnaporthales genome and transcriptome data, we set up an FTP site (ftp://172.21.7.71/pub/Magnaporthales/) that includes the assembled genomes and transcriptomes of the five species sequenced for this project, transcriptomes from an additional 16 Magnaporthales species^9^, and the genomes and transcriptomes of eight species that were downloaded from public databases and used in our work. Genome assemblies, annotations, and associated raw sequence reads from this study were submitted to GenBank (BioProject #: PRJNA438993). Using the genome data from the 13 Sordariomycetes species, a total of 14,835 gene families were generated using OrthoFinder that are present in at least two different species. Using a phylogenomic approach, we generated phylogenetic trees for all orthologous gene families and singleton genes. The orthologous gene families and the phylogenetic trees (with ≥5 sequences) are available for download from the FTP site and were used for downstream analysis.

### Estimating a timeframe for Magnaporthales diversification

**Figure 1:**
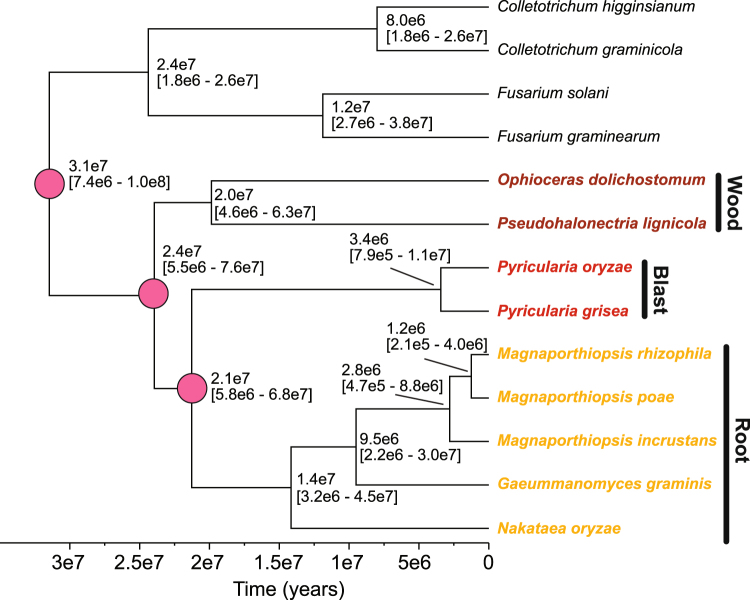
Rooted maximum credibility tree constructed from 1000 single copy orthologous proteins using BEAST. The bootstrap support values were 100 on all branches. Node labels show mean node ages, and 95% highest posterior density intervals. The three clades of Magnaporthales are indicated as are their split dates (with pink filled circles).

We generated a Bayesian maximum credibility tree with mean node age inferred using BEAST, based on 1000 randomly selected single copy orthologs. This phylogeny is shown in Fig. 1. The six replicate MCMCs converged on all parameters, posteriors, and likelihood estimates. The effective sample size values were all >800, and the topology of the tree was identical to the topology of the maximum likelihood tree inferred from the concatenation of the 1000 randomly selected orthologs using RAxML v.8.2.4^10^ (not shown). Only results of the run with the highest posterior probability are presented. All nodes had 100% Bayesian posterior support. The estimates of divergence time indicate that the Magnaporthales diverged ca. 31 million years ago (95% highest posterior density interval [95%HPD]: 7.3e6–10e8) from the group formed by *Colletotrichum* spp. and *Fusarium* spp. (Fig. 1). The split between the wood and blast/root clade occurred about 24 million years ago with the blast clade splitting off about 21 million years ago.

It should be noted, that divergence times estimated from molecular clock analyses largely depend on the values used to define the prior distribution on the substitution rate. In our analysis, the minimum, maximum, and average values of the prior distribution on the substitution rate were defined based on previous estimates of fungal nucleotide substitution rates obtained using different calibration time points (protein clock-based estimate of fungi-animal divergence^11^, protein clock-based estimate of fungi-plant-animal divergence^12^, and ascomycete fossils)^13^. These bounds include the estimate of the substitution rate in *P. oryzae* inferred from a tip-calibrated genome genealogy^14^. The minimum and maximum values were also within the range of substitution rates in unicellular eukaryotes^15^. Because we used plausible substitution rates and relied on a molecular clock framework that accounted for rate variation along sequences and among lineages, we are confident that our divergence time estimates represent a reasonable approximation for the timeframe of Magnaporthales diversification.

### Extent of horizontal gene transfer in Magnaporthales

To assess if HGT may have played a role in the origin of genetic innovations in the studied 13 species of Sordariomycetes, we calculated the Alien Index (AI)^16,17^ for all protein coding genes in each genome. Briefly, for each gene, we compared the strength of its best sequence match in eukaryotes (not including other Sordariomycetes) to its best match in prokaryotes (see Methods). This procedure resulted in a preliminary gene set (516 in total, 0.39%) with significantly stronger hits to prokaryotes than eukaryotes (AI ≥0.05). Depending on the species, the number of genes ranged from 0.24% (in *P. lignicola*) to 0.55% (in *F. solani*). These percentages are similar to those reported in an earlier analysis of bacterium-derived genes in fungi^18^. We also examined the corresponding phylogenetic trees generated from our conservative phylogenomic approach described above. A total of 195 cases with conflicting phylogenies (mostly representing more ancient gene transfers earlier than Sordariomycetes ancestor) were filtered out. The remaining 321 cases have trees that are either supportive or inconclusive (regardless of branch support), making them candidates for further investigation (Table S4).

**Figure 2:**
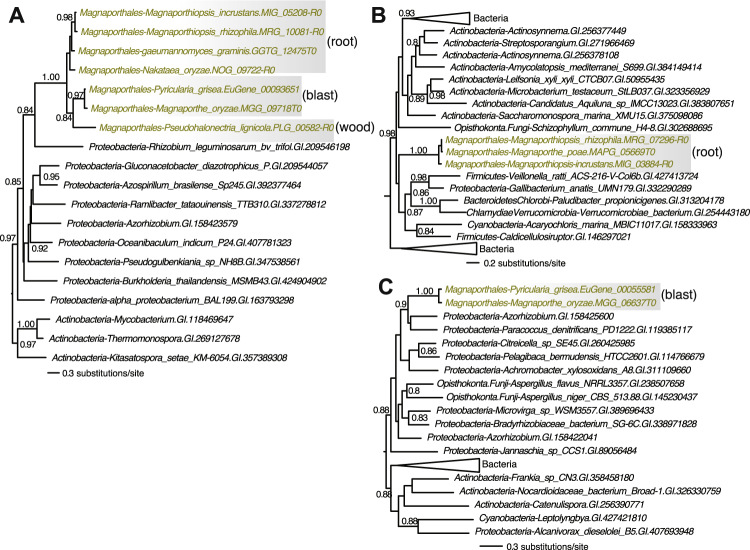
Bacterial-derived HGTs in Magnaporthales. (**A**) Phylogenetic tree of a fumarylacetoacetate hydrolase. (**B**) Phylogenetic tree of a N-acetyltransferase family protein. (**C**) Phylogenetic tree of a N-acetyltransferase family protein. Statistical support (>0.8) is indicated for each branch. Magnaporthales are shown in brown text and prokaryotes in black text. The major clades impacted by HGT (i.e., wood, blast, root) are shown.

We then focused on HGTs that are specific to the Magnaporthales. A total of five bacterium-derived HGTs were identified that were supported by both the AI-based and phylogenomic methods (Figs 2, S1). These include a gene encoding fumarylacetoacetate hydrolase (FAH, Fig. 2A) that is present in all three Magnaporthales major lineages [wood, blast, and root (Luo *et al*.^9^)]. This gene was most likely transferred into the Magnaporthales common ancestor. FAH participates in amino acid (tyrosine) catabolism hydrolyzing fumarylacetoacetate into fumarate and acetoacetate. We also found the transfer of a gene encoding N-acetyltransferase family (pfam00583) protein into the Magnaporthales root clade (Fig. 2B). Interestingly, another bacterial gene of the same family was transferred independently into the Magnaporthales blast clade (Fig. 2C). Although these two transferred proteins share limited sequence identity (<30%), it is likely that a similar function was independently acquired in these two instances. The remaining two HGTs represent an antibiotic biosynthesis monooxygenase and a hypothetical protein (Fig. S1). Taken together, the small number of HGTs found that are specific to Magnaporthales is consistent with the overall paucity of cross-phylum gene transfers in this lineage^19^. Given our focus on a conservative HGT dataset and extensive intra-phylum gene exchanges that may compromise HGT detection^19^, these HGTs likely represent a subset of genes that were transferred into Magnaporthales. The existence of clear evidence of bacterium-derived genes suggests that HGT might have played an important role in Magnaporthales evolution and function.

### Branch-site selection tests

**Figure 3:**
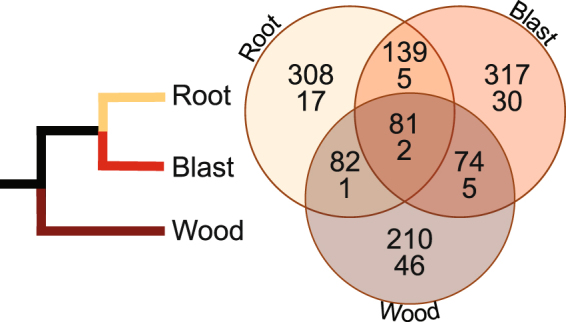
Schematic phylogeny (left image) of the three major Magnaporthales clades proposed by Luo *et al*.^9^ The right image is a Venn diagram showing shared and unique ortholog groups (OGs, top number) and gene ontology terms (GO terms, bottom number) that show evidence of positive selection among the three lineages.

Beyond HGT, genetic innovation can also result from positive selection acting on fungal genes in response to environmental constraints or life cycle evolution. To address this issue, we did enrichment tests on the genome data (see Methods). This analysis identified 447, 611, and 610 ortholog groups (OGs) that show evidence of positive selection (FDR ≤0.01) in the wood, blast, and root infecting fungal clades^9^ (Table S5). Blast2GO enrichment analyses highlighted 54, 42, and 25 over-represented “most specific” gene ontology (GO) terms in each group, respectively (Tables S6, S7). Examination of this list allowed us to identify shared and lineage-specific GO terms and OGs that shed light on particular genes and gene families and molecular mechanisms responsible for phenotypic and phylogenetic diversification of the Magnaporthales fungal clades. Only 81 of the 1,211 total OGs showing evidence of positive selection were shared in all three clades tested; the majority are specific to individual clades (Fig. 3). The root and blast fungal clades shared the highest number of OGs, likely owing to their common ancestry^9^. Particular attention was given to enriched GO terms from within individual wood, blast, or root clades comprised predominantly of OGs not present in the remaining two groups (Tables S6, S7), because these would represent clade- or lineage-specific targets of selection. We focused on these terms for the remaining discussion below.

Gene ontologies enriched in the blast clade include those involved in DNA repair (GO:0003684 *damaged DNA binding*, GO:0019985 *translesion synthesis*, GO:0035267 *NuA4 histone acetyltransferase complex*), signaling and response to stimulus (GO:0009607 *response to biotic stimulus*, GO:0046578 *regulation of Ras protein signal transduction*), malic enzyme activity (GO:0006108 *malate metabolic process*, GO:0016615 *malate dehydrogenase activity*), glutamate synthase/dehydrogenase activity (GO:0009064 *glutamine family amino acid metabolic process*, GO:0016639 *oxidoreductase activity, acting on the CH-NH2 group of donors*, *NAD or NADP as acceptor*), synthesis and catabolism of small molecules (GO:0046165 *alcohol biosynthetic process*, GO:0044282 *small molecule catabolic process*) along with genes involved in septation during cytokinesis^20^ (GO:0005826 *actomyosin contractile ring*). The root infecting fungal branch was enriched for ontologies primarily associated with peptidases (GO:0004222 *metalloendopeptidase activity*, GO:0004252 *serine-type endopeptidase activity*), small molecule metabolic processes (GO:0009132 *nucleoside diphosphate metabolic process*, GO:0015940 *pantothenate biosynthetic process*), transferases (GO:0016409 *palmitoyltransferase activity*, GO:0016757 *transferase activity, transferring glycosyl groups*, GO:0070566 *adenylyl transferase activity*), isomerases (GO:0016853 *isomerase activity*) as well as gene families involving the function and morphology of the fungal-type vacuole (GO:0000324 *fungal-type vacuole*), a lytic membrane-bound compartment that functions in degradation and also stores small molecules^21^.

The more diverged and niche-adapted saprotrophic wood-inhabiting fungi showed evidence of positive selection on the largest number of ontologies, including those involved in chromatin and chromosome modification (GO:0000083 *regulation of transcription involved in G1/S transition of mitotic cell cycle*, GO:0000414 *regulation of histone H3-K36 methylation*, GO:0035327 *transcriptionally active chromatin*, GO:0016233 *telomere capping*, GO:0000118 *histone deacetylase complex, GO:0051568 histone H3-K4 methylation*), transcription and RNA processing (GO:0098781 *ncRNA transcription*, GO:0032545 *CURI complex*, GO:0006409 *tRNA export from nucleus)*, the (endo)membrane system (GO:0005643 *nuclear pore*, GO:0017056 *structural constituent of nuclear pore*, GO:0032541 *cortical endoplasmic reticulum*, GO:0090158 *endoplasmic reticulum membrane organization*, GO:0060304 *regulation of phosphatidylinositol dephosphorylation* [phosphatidylinositol is a key membrane constituent]), the spliceosomal complex (GO:0000244 *spliceosomal tri-snRNP complex assembly*, GO:0005682 *U5 snRNP*), and organelle and cytoskeletal organization (GO:0034631 *microtubule anchoring at spindle poll body*, GO:0036257 *multivesicular body organization*).

We also examined the seven over-represented GO terms shared between the root and blast pathogenic fungal clades (Table S6), because these may comprise common gene families that elucidate pathogen adaptation. Of the seven terms, six (GO:0030422 *production of siRNA involved in RNA interference*, GO:0031048 *chromatin silencing by small RNA*, GO:0031332 *RNAi effector complex*, GO:0051570 *regulation of histone H3-K9 methylation*, GO:0031618 *nuclear pericentric heterochromatin*, GO:0033562 *co-transcriptional gene silencing by RNA interference machinery*) contain a common group of enzymes (Argonaute-binding 1, dicer 1, piwi domain-containing) that implicate the RNA interference pathway, whereas the final term (GO:0031573 *intra-S DNA damage checkpoint*) describes a pathway that slows DNA synthesis in response to DNA damage^22^. Both RNAi and small RNAs have recently been shown to facilitate infection of plant hosts by fungal pathogens^23^, and components of the DNA damage checkpoint also appear to function in the switch from unicellular growth to the hyphal stage (the infectious stage in pathogenic fungi) in *Schizosaccharomyces* species^24^. These particular GO terms may therefore highlight drivers of differentiation and adaptation in fungal pathogens. We analyzed the 139 OGs shared between the root and blast clades that show evidence of selection and extracted the most abundant GO term from the annotations (irrespective of statistical representation). This term was GO:0016021 *cellular component of membrane* (28 occurrences), indicating membrane-anchored components are widespread in this subset of genes.

### Secretome and effectors in Magnaporthales

Moving from the molecular evolution of pathogenic Magnaporthales genomes, we next focused on the secretome of this clade and encoded effectors. The results of this analysis for the sizes of the secretome and small secreted proteins (SSPs), and species-specific SSPs in Magnaporthales species (SSSPs) are listed in Table 1 and the identifiers are provided in Table S8. The clade-specific secretomes and SSPs were also identified (see Table 2, identifiers in Table S9). The two species in the blast pathogen clade, *P. grisea* and *P. oryzae* had the highest number of secretome, SSPs, and SSSPs when compared to the other Magnaporthales species. The blast pathogens also had remarkably more clade-specific secretomes (454) and SSPs (385) than the root pathogen clade (15 and 12 respectively) and the wood non-pathogen clade (55 and 31).

**Table 1:** Predicted secretome size in different Magnaporthales species.

Species	Secretome	SSPs	SSSPs
*G. graminis*	1,204	545	91
*P. grisea*	1,369	743	143
*P. oryzae*	1,426	772	221
*M. poae*	1,076	565	58
*M. incrustans*	1,203	528	54
*M. rhizophila*	1,136	490	29
*N. oryzae*	1,075	460	81
*O. dolichostomum*	1,022	432	110
*P. lignicola*	1,038	410	93

**Table 2:** Clade-specific secretomes and small secreted proteins (SSPs).

Clade	Secretome	SSPs
**Blast pathogen clade**	454	385
*P. oryzae*	227	191
*P. grisea*	227	194
**Root pathogen clade**	15	12
*M. poae*	4	4
*M. incrustans*	4	4
*M. rhizophila*	3	2
*N. oryzae*	4	2
**Wood (non-pathogen) clade**	55	31
*O. dolichostomum*	29	15
*P. lignicola*	26	16

**Figure 4:**
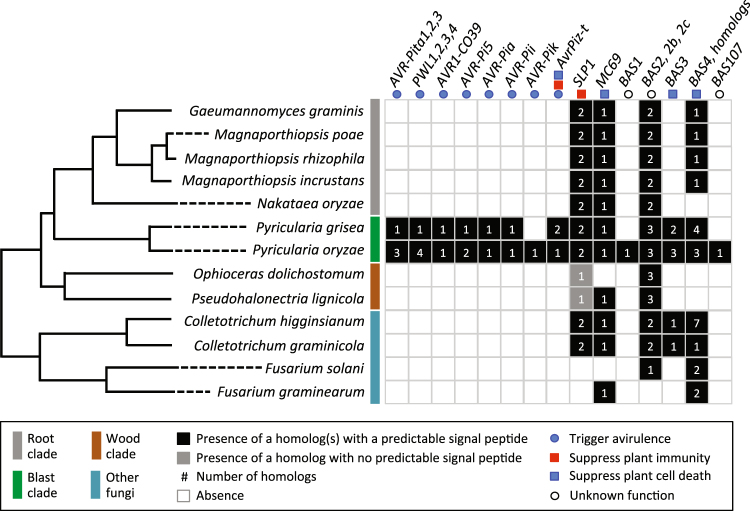
Distribution of different effectors in the Magnaporthales showing the existence of both clade-specific and more widely conserved genes.

Our analysis of effectors revealed both clade-specific effectors and ones that are conserved across multiple clades with a varying number of homologs (Fig. 4). The clade-specific effectors include eight avirulence genes that are exclusively present in various strains of *P. oryzae* and *P. grisea*, belonging to the blast clade. Increasing evidence suggests that avirulence genes can also play roles in infection of susceptible hosts^25,26^. For example, an avirulence gene *AvrPiz-t* encodes a secreted protein that enters the host cytoplasm, where it interacts with the rice RING E3 ubiquitin ligase APIP6 to suppress PAMP-triggered immunity (PTI). Given the blast clade-specific occurrence of these avirulence genes, their known and presumed roles are likely to be specific to the interactions between *Pyricularia* species and their hosts.

Another host PTI suppressor, Slp1, has been characterized in *P. oryzae*. Slp1 is a secreted LysM protein that suppresses host PTI by scavenging PAMP molecules such as chitin in the apoplast, thus being required for *P. oryzae* virulence^27^. LysM proteins are highly conserved across fungi, and in particular, Slp1 shows strong similarity to the Ecp6 chitin binding protein found in *Cladosporium fulvum*^27^. Not surprisingly, we found that Slp1 homologs were present in all Magnaporthales and *Colletotrichum* species (Fig. 4). Interestingly, the wood clade fungi carry one Slp1 homolog that lacks a predictable signal peptide, suggesting that this protein may have a divergent function than other secreted Slp1 homologs. Three effectors suppress plant cell death^28^, and are required for *P. oryzae* to establish biotrophy at the early stage of rice infection. These effectors include MC69 (MGG_02848), which is essential for full virulence of *P. oryzae* and is conserved in many plant pathogenic fungi^29^, and two Bas proteins Bas3 (MGG_11610) and Bas4 homologue (MGG_02154) (Fig. 4).

**Figure 5:**
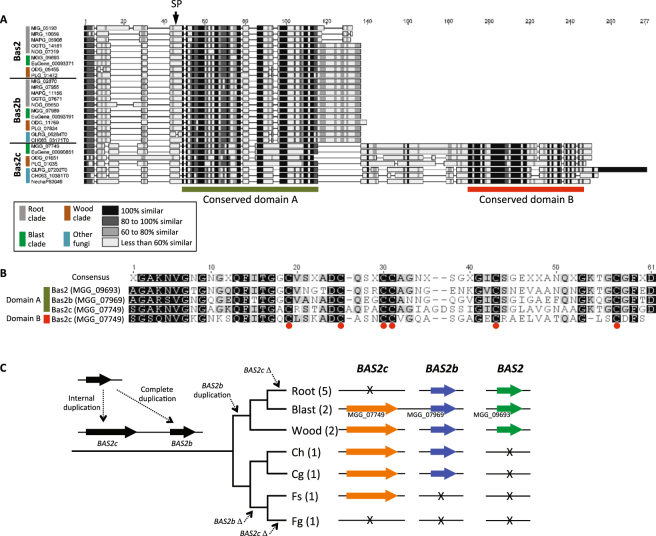
Analysis of Bas proteins in the Magnaporthales. (**A**) Domain structure between gene family members. (**B**) Alignment showing conserved residues. (**C**) Phylogenetic distribution of Bas proteins and history of gene/domain duplication among the three major Magnaporthales clades.

*BAS2* (MGG09693) belongs to a multigene family with two other members^30^, named here as *BAS2b* (MGG07969), and *BAS2c* (MGG07749). We found that all sequenced genomes included in this study, with the exception of *F. graminearum*, encode at least one member of the family (Fig. 4; Tables S8, S9). The wide occurrence of the *BAS2* family suggests its role in fungal adaption to diverse hosts and environments. To gain insights into the role of these effectors in fungal adaptation, we reconstructed the evolution of the *BAS2* family by analyzing the presence/absence polymorphism and sequence similarity of the family members. Our analysis revealed that *BAS2* and its orthologs were present only in Magnaporthales, suggesting they were derived from Magnaporthales-specific gene duplication. The presence of the orthologs of *BAS2b* and *BAS2c* in *Colletotrichum* species and their absence in some lineages support their ancestral presence and subsequent loss in respective lineages. In the sequenced genome of *P. oryzae* strain 70–15, *BAS2* is located on chromosome IV, whereas *BAS2b* and *BAS2c* are located ~800 kb apart on chromosome III. Chromosomal locations of *BAS2* homologs found in other sequenced genomes remain undetermined due to gaps in genome assemblies. Interestingly, *BAS2c* and its orthologs encode larger-secreted proteins, ranging from 173 to 204 residues, than those of *BAS2* (86–101 residues) or *BAS2b* (99–108 residues). A dot plot analysis and sequence comparison revealed that the Bas2c protein contained two domains that share high sequence similarity with each other and also six conserved Cys residues in each domain (Fig. 5; Tables S8, S9). These data suggest that *BAS2c* resulted from an internal gene duplication, which likely led to the gain of a new function, potentially providing a beneficial adaptation^31^. Consistent with this idea, targeted gene replacement of *BAS2* does not result in pathogenicity phenotypes^30^, possibly due to functional redundancy with its duplicate, *BAS2b*. We propose that the *BAS2* gene family has undergone both internal and complete gene duplications to generate functionally divergent family members as well as multiple losses during fungal adaptation to diverse habitats.

### Analysis of transposable elements

Each of the nine genomes, plus that of *Falciphora oryzae* (syn. *Harpophora oryzae*), were individually checked for TEs using the REPET vr. 2.5 pipeline. The combined length of all TE matches identified by REPET as a percentage of genome size is shown in Fig. S2A. These results ranged from 0.64% (253,718 bp) for *M. poae* to 15.35% (7,795,925 bp) for *F. oryzae*. We note the proportion of TEs shown is approximately the same as the numbers previously reported by Xu *et al*.^32^ for *F. oryzae* (+0.4%), *P. oryzae* (+1.7%), *M. poae* (−0.01%) and *G. graminis* (−0.3%) (Fig. S2A). These small differences in the overall proportion of TEs likely stem from Xu *et al*. using a different program, RepeatModeler^33^, for their TE prediction protocol. Class I TEs (retrotransposons) were found to be more abundant than Class II TEs (DNA transposons) across all genomes. The ratio of Class I to Class II TE percentages shown ranges from 2:1 for *M. oryzae* to almost 20:1 for *M. rhizophila* (Fig. S2A).

The relative abundance of TEs as a percentage of genome size, when grouped by TE order is shown in Fig. S2B. Long terminal repeat (LTR) TEs were found to be the most abundant Class I order for all surveyed genomes. Consistent with previous reports^6^, the LTR retrotransposons, were the most abundant order of TEs in the genome of *M. oryzae* (3.3% of all TEs), but our analysis showed that members of the DIRS family were found in nearly as high abundance (3.2% of all TEs). Amongst Class II orders, terminal inverted repeats (TIRs) were the most abundant for all genomes with the exception of *M. incrustans*, which encoded more Helitrons. At the superfamily level, Gypsy was generally the most abundant TE, followed by Copia. Analysis of novel TEs and evidence of repeat-induced point mutation (RIP) are described in the Supplementary Text.

TEs have been shown as important drivers of genome evolution in the several species of Magnaporthales^6,32,34–36^. For example, in *F. oryzae* TEs have played a major role in the lifestyle transition from parasite to beneficial endophyte during rice root infections by causing species-specific gene expansions^32,37^. The species-specific nature of these TE expansions in the Magnaporthales can be observed in Fig. S2A, where the percentage of the genome occupied by TEs varies greatly. Even amongst *F. oryzae, G. graminis*, and *M. poae*, which all infect rice roots and share high levels of macrosynteny^32^, both the lowest and highest TE percentages in Fig. S2A are seen with *M. poae* and *F. oryzae* (a difference of 14.7%). Whereas environmental differences amongst all taxa likely explains much of the variability in the number of TEs present, the role of RIP in limiting TE expansion is another factor that is relevant in the genome of *M. oryzae*^6^. RIP was found present in almost 80% of the TE superfamilies assessed in Fig. S3 and in at least one family for all genomes considered except for *O. dolichostomum* and *M. rhizophila*. One possibility for RIPs not being identified in the latter two species is the large number of unclassified LTR, LINE, and TIR sequences found using REPET. Collectively, these unknown TEs account for 1.3% and 5.8% of the *O. dolichostomum* and *M. rhizophila* genomes, respectively. Accordingly, many of these sequences may belong to TE superfamilies that did not meet the quantity or length requirements used to test for RIP using RIPCAL. Amongst the TEs identified across genomes, Class I members were found to consistently outnumber those in Class II. LTRs and TIRs were generally found to be the most prevalent Class I and Class II TE orders, respectively. Finally, analysis of REPET *de novo* predicted TE families identified many clusters with high similarity to known AVR genes in *M. oryzae*. This lends further support to the importance of TEs in shaping fungal-plant interactions amongst the Magnaporthales.

## Conclusions

Our study provides a genome-based perspective on the evolution of Magnaporthales, suggesting that this order diverged from other Sordariomycetes about 31 million years ago with the phytopathogenic blast clade diverging ca. 21 million years ago. We find little evidence of HGT among these taxa, with only a handful of *bona fide* candidates, although intra-phylum gene movement may be much higher but was not the target of our conservative phylogenomic approach. In contrast, many more genes underwent positive selection in Magnaporthales with only 81/1,211 OGs in this category being shared by all three clades. The majority are clade-specific, with the wood clade having the largest number of gene ontologies impacted by positive selection. Analysis of the secretome and effectors showed that the blast clade is most enriched in the latter as avirulence genes. These likely play key roles in the interaction between *Pyricularia* species and their plant hosts. Finally, analysis of TEs showed different proportions of TE classes in the Magnaporthales genomes, suggesting that species-specific patterns may hold clues to the history of host/environmental adaptation in these lineages. We did not include in this manuscript an analysis of biosynthetic pathways of secondary metabolites in Magnaporthales. Nonetheless, we recognize that these gene clusters are of significant interest to mycologists, in particular, the comparison of pathways in pathogenic and non-pathogenic fungi. We hope that the genome data produced here will enable investigation of this and other important topics in the near future. In summary, our comparative analyses point out the divergent histories of fungi in the important Magnaporthales order and provide clues to how these taxa have adapted to their vastly different niches in the past 31 million years.

## Methods

### Genome sequencing, assembly and annotation

Five species in Magnaporthales, *M. incrustans* (strain M35)*, M. rhizophila* (strain M23)*, N. oryzae* (strain M69)*, O. dolichostomum* (strain CBS114926) and *P. lignicola* (strain M95) were subjected to genome and transcriptome sequencing in this project. For genome sequencing, DNA was extracted from mycelium after 14 days of growth on potato dextrose agar (PDA) at 25 °C using DNeasy Plant Mini Kit (Qiagen, CA). A Nextera DNA library with average insert size approximately 450 bp was constructed for each species following manufacturer’s instructions (Illumina). The libraries were sequenced to 2 × 146 bp on GenomeAnalyzer IIX (Illumina). For transcriptome sequencing, strains were grown at 25 °C on four different conditions: PDA under 12/12 hours light-dark cycle, PDA in constant darkness, minimal medium (MM, 10 g sucrose, 1 g Ca(NO_3_)_2_·4H2O, 0.2 g KH_2_PO_4_, 0.25 g MgSO_4_·7H_2_O, 0.15 g NaCl, and 15 g agar in 1 liter distilled water) under 12/12 hours light-dark cycle, MM in constant darkness. A cellophane membrane was placed on top of the media. After 14 days of incubation, the mycelium was harvested and total RNA extracted from each sample using the Plant RNeasy Mini Kit (Qiagen, CA). A TruSeq mRNA library was constructed for each sample following manufacturer’s instructions (Illumina). The libraries were sequenced on MiSeq (Illumina) to 2 × 158 bp.

Adapter sequences were trimmed from sequence reads of genomic DNA using Trimmomatic^38^. Trimmed reads were corrected using the correction module in SOAPdenovo2 r239 package^39^ with k-mer size 21. Corrected reads were assembled using SOAPdenovo2. A range of k-mer sizes were tested and optimal k-mer sizes were choosen for individual genomes. Scaffolds and singleton contigs less than 500 bp in length were filtered out from final assemblies. To test the completeness of assembled genomes, CEGMA (Core Eukaryotic Genes Mapping Approach)^40,41^ analysis was used to look for the 248 conserved eukaryotic genes in assembled genomes.

The assembled genomes were annotated using the MAKER2 package^42^ with three gene prediction tools used: AUGUSTUS^43,44^, SNAP^45^ and the *ab initio* tool GeneMark-ES version 2^46^. AUGUSTUS was trained with *M. oryzae*, and SNAP was initially trained with CEGMA results. MAKER2 was run for four iterations for each genome. Gene models from the previous run were used for re-training in the following run. Adapter-trimmed transcriptome sequences were used to assemble EST sequences with TRINITY^47^ using the assembled genomes as guide. The results were used as EST evidences in MAKER2 runs. Proteome sequences of *G. tritici* strain R3-111a-1, *M. oryzae* strain 70–15_8 and *M. poae* strain ATCC64411, and the manually annotated Swiss-Prot^48^ protein sequences were used as protein evidences. The flanking regions were set to 50 bp to mitigate gene model fusion which could occur in compact fungal genomes. After four iterations, the optimal gene models chosen by MAKER2 from the three gene prediction tools were used for sownstream analysis.

### Sequence availability

Genome assembly, transcriptome and proteome sequences of eight species were downloaded from public databases. Those of *C. graminicola* strain M1.001, *C. higginsianum* strain IMI 349063, *F. graminearum* strain PH-1, *G. tritici* strain R3-111a-1, *M. oryzae* strain 70-15_8 and *M. poae* strain ATCC64411 were downloaded from Broad Institute (http://www.broadinstitute.org/ftp/pub/annotation/fungi/); those for *F. solani* was obtained from ENSEMBL (ftp://ftp.ensemblgenomes.org/pub/fungi/release-30/fasta/fusarium_solani/); and files for *P. grisea* strain BR29 were obtained from INRA (http://genome.jouy.inra.fr/gemo/).

### Construction of orthologous gene families

We collected proteome data from the 13 Sordariomycetes species described above. After removing short sequences (<50 amino acids), a total of 171,033 sequences were used to construct orthologous gene families using OrthoFinder version 0.4^49^ under the default setting (BLASTp e-value cut-off = 1e-5; MCL inflation I = 1.5). A total of 14,835 gene families were generated that are available at our FTP site (ftp://172.21.7.71/pub/Magnaporthales/).

### Timing of Magnaporthales diversification

Divergence time estimates were based on a subset of 1000 orthologs randomly selected (using python package random) from the full set of 3,060 single copy orthologs. The 3,060 groups were selected by parsing the ortholog groups (OGs) derived above, and keeping only single-copy genes (i.e., had no evidence of duplication) in all 13 taxa. Sequences were aligned using MACSE^50^, with default parameters. MACSE alignments were processed to remove all codons including sites with a gap in at least one of the samples, and then concatenated. Node ages were estimated using a Bayesian molecular clock model implemented in the program BEAST v1.8.0^51,52^. The analysis used a General Time Reversible model (GTR) with Gamma distribution for modeling site heterogeneity and base frequencies estimated from the data. The clock model was a random local clock, and the coalescent tree prior assumed an unknown constant population size back through time. We specified the normal prior distributions on substitution rates. We used the minimum and maximum values of exonic substitution rates estimated by Kasuga *et al*.^53^ using fossil-calibrated phylogenies as the lower and upper bounds (0.13e-9 and 1.759e-8 substitutions/site/year, respectively). The initial value and the mean were set at 8.86e-9 substitutions/site/year [(0.13e-9 + 1.759e-8)/2], and the standard deviation was 1e-8. We edited the XML file generated by BEAUti v.1.8.2. to estimate the age of all nodes in the tree. We used UPGMA tree as the starting tree and ran six independent MCMC of 7.5e7 steps to ensure convergence, logging parameters and trees every 7500 steps. The program Tracer v1.5^52^ was used to evaluate all chains, determine appropriate burn-in length (10% of sampled trees), and obtain the effective sample sizes for all parameters. Mean node age and 95% highest posterior density were mapped on the maximum clade credibility tree.

### Screen for HGT candidates

For each Sordariomycetes genome, all predicted proteins were queried against NCBI’s non-redundant protein database (November 10, 2015) using ublast with database seq masking enabled, dbaccel parameter of 100, accel parameter of 1, and e-value cutoff of 0.001 (http://drive5.com/usearch)^54^. A modified alien index approach^16^ was used to screen each genome for genes with significantly better ublast hits to prokaryotes than eukaryotes and thus identify trans-domain HGT candidates. The AI score for this analysis is given by the formula:

$$AI=(\frac{bbhO}{maxB}-\frac{bbhE}{maxB}),$$

where *bbhO* is the bitscore of the best blast hit to a species outside of the eukaryotic lineage, *bbhE* is the bitscore of the best blast hit to a eukaryote, and *maxB* is the maximum bitscore possible for the query sequence (i.e., the bitscore of the query aligned to itself). To identify horizontally transferred genes shared between multiple species of Sordariomycetes (e.g., HGT that occurred in a common ancestor), all hits to Sordariomycetes were skipped and did not contribute to *bbhE*. *AI* can range from 1 to −1, and *AI* >0 if the gene has a better blast hit to a species outside of the eukaryotic lineage and is suggestive of either HGT or contamination. Contamination was suspected in cases when the majority of genes on an assembly contig had an *AI* ≥0; in contrast, HGT was inferred when the transferred gene was located within a well assembled contig, and the majority of genes on that contig had an *AI* <0^55^. The perl script for calculating AI scores is freely available from the figshare repository, 10.6084/m9.figshare.1593040.

### Phylogenomic analysis

To understand the evolutionary history of each Sordariomycetes gene, we carried out phylogenomic analysis in a similar way to our previous study^19^. Briefly, a local database was established containing Pezizomycotina sequences from NCBI RefSeq database (version 69) and the sequences for the remaining species from RefSeq database (version 55)^19^. For each Sordariomycetes orthologous gene family and singlet, we used the corresponding protein sequence(s) as query to search the above-mentioned local database using BLASTp (e-value cut-off = 1e-5). For each singlet query, the top 1,200 significant hits (with query-hit identity ≥30%) were recorded followed by selection of representative sequences in order. Up to 10 sequences were sampled from each phylum. The sequence sampling ceased when a total of 80 sequences were selected or the entire list of significant hits was read through. For the gene families containing two or more members, the quota for sequence sampling was divided among members and the sampled sequences were pooled. When the sampled unique sequences were less than 80, further rounds of sequence sampling were carried out until more than 80 unique sequences were sampled. The retrieved sequences were combined with query sequences and were then aligned using MUSCLE version 3.8.31^56^ under default settings followed by trimming using TrimAl version 1.2^57^ in an automated mode (−automated1). We removed columns with >50% gaps and the resulting alignments were then used to build phylogenetic trees using FastTree^58^ under the WAG + CAT model with four rounds of minimum-evolution SPR moves (−spr 4) and exhaustive ML nearest-neighbor interchanges (−mlacc 2 −slownni). Branch supports were calculated using the Shimodaira-Hasegawa method^59^.

### Branch-site selection tests

The corresponding protein sequences that composed the OG were aligned with MAFFT v7^60^ and a codon alignment for each OG was then created with TranslatorX^61^ using the nucleotide CDS sequence and aligned protein data as a guide. MaxAlign v1.1^62^ was used in codon mode to remove poorly aligned sites and sequences. Any taxa removed from the OG by MaxAlign were then removed from the phylogenetic tree (above) using PareTree (http://emmahodcroft.com/PareTree.html). The branch-site selection test implemented in codeml (part of the PAML package v.4.8a)^63^ was then used to test for positive selection on three branches of the phylogeny leading to the “wood”, “blast” and “root” fungi (see above)^9^. If all taxa within a particular clade (i.e. both *Pseudohalonectria lignicola* and *Ophioceras dolichostomum* or “wood” saprophytic fungi)^9^ were removed by PareTree, the OG was ignored for that particular test. The log-likelihoods generated by codeml under a null hypothesis of neutral evolution (omega fixed at 1) and the alternative (ML estimate of omega) were compared using a likelihood ratio test to a chi-square distribution (df = 1) and false discovery rate (FDR) tests were performed in R using the Bioconductor package. Ortholog groups with a corresponding test statistic (FDR ≤0.01) were retained. Gene Ontology terms were assigned to each gene in each OG using Blast2GO^64^, and annotated further against the NCBI nr database using BLASTp. A Fisher’s exact test was conducted in Blast2GO to find enriched (or over-represented) GO terms (*p* ≤ 0.05) within the set of OGs found to be under positive selection as compared to a reference set consisting of all 3,060 OGs. GO terms were condensed to “most specific terms”, i.e. less descriptive parent terms in the hierarchy were removed and only the most-descriptive child terms were retained. If an OG was excluded from the “wood”, “blast” or “root” analysis based on MaxAlign criteria, it was also removed from the reference.

### Whole-proteome MCL clustering analysis

The whole-proteome clustering analysis for 9 Magnaporthales spp. and 4 closely related species was performed using the MCL algorithm (http://micans.org/mcl/)^65^. The parameters used for clustering were the same as for orthologus gene group prediction (BLASTp: *E* = 1e-5, MCL clustering inflation = 1.5).

### Secretome analysis

The secretome of the nine Magnaporthales species were predicted using the pipeline described in Kim *et al*.^66^ and the number of small secreted proteins (SSPs, <300 aa) were counted. The pipeline strictly excludes membrane-bound secreted proteins (both transmembrane helix containing and GPI-anchored proteins); therefore, the predicted secretome sizes are generally smaller than the ones only predicted with signalP Eukaryotes. The number of SSPs were further reduced by comparing to above species-specific clustering data to find the ones with effector-like property, which are referred as SSSPs. Based on the whole protein OrthoFinder clusters and the secretome results, clade-specific secretomes were also identified.

Many plant pathogenic fungi secrete effector proteins that facilitate disease development and modulate plant immunity. However, avirulence effectors trigger defense responses in hosts with cognate R genes and block disease. From various strains of *P. oryzae*, over 200 effector and effector candidate genes have been identified, some of which belong to multigenic families^28,67^. Of these, we selected 28 well-characterized effector genes and their homologs to survey for their presence in 13 sequenced genomes of nine Magnaporthales species and four closely related fungal pathogens including *Colletotrichum* species and *Fusarium* species.

### TE identification

Each of the nine Magnaporthales genomes, plus the genome for *Falciphora oryzae*, were searched for TEs using the REPET vr. 2.5 pipeline^33^ according to the authors’ documentation (https://urgi.versailles.inra.fr/Tools/REPET). The pipeline begins with the *TEdenovo* script, which uses a series of classifiers to identify a set of *de novo* TEs which are combined to form a database for use with the second *TEannot* script. This latter stage is run twice in order to perform a complete genome-wide annotation using multiple classifiers. A step is also performed which employs tBLASTx and BLASTx from the BLAST + vr. 2.2.31 package^68^ to search Repbase vr. 20.05^69^, a curated data bank of TEs. Both stages were run using default settings and also incorporated the Pfam vr. 27.0^70^ data bank. After identifying TEs throughout the genome, final annotation was exported as a GFF3 file using an option to merge overlapping TE entries. Counts of TE superfamilies were made for each genome using these files according to the Wicker classification scheme^71^. According to recommendations by Wicker *et al*.^71^, sequences less than 80 bp in length were excluded to avoid misclassification. Other TEs removed included potential host genes and predictions made from tBLASTx and BLASTx with less than 70% sequence identity reported. As some genomes had many hits classified by family and order, but listed as “unknown” for superfamily (e.g. *DTX*, an unknown Class II terminal inverted repeat), we ran an additional tBLASTx search (e-value: 0.01, percent query coverage per hsp: ≥70%, minimum percent identity of hits: ≥70%) against Repbase for these sequences. If a top hit matching the above search criteria had the same family and order as the original REPET prediction, we reassigned the superfamily based on the matching Repbase entry. Additional details about the REPET pipeline can be found in Flutre *et al*.^33^.

### Cross-genome comparison of REPET *de Novo* TEs

Similarity across genomes for TEs predicted by the REPET *TEdenovo* pipeline (excluding incomplete and potential host gene sequences) was checked using hierarchical clustering with the *h-cd-hit-est* program from the CD-HIT Suite Server (http://weizhongli-lab.org/cd-hit/)^72^. The 580 initial sequences were grouped (reverse compliments considered) using iterative cutoffs of 90%, 85% and 80% sequence identity to form 347 clusters. Custom scripts were then used to identify the number of different taxa present in each cluster based on sequence definitions. Representative sequences for each cluster were then blasted against the NCBI nucleotide (nt) database using Blast2GO^64^ for annotation.

### Repeat-induced point mutation identification

TE families for each genome were searched for signatures of RIP activity using the RIPCAL vr. 2 program^73,74^. The program was only used to check for RIP within individual TE families which contained a minimum of ten sequences, with one or more sequence ≥300 bp (the default RIPCAL scanning subsequence length), using both the alignment with ClustalW^75^ and di-nucleotide frequency-based methods. These cutoff criteria were established to minimize computational requirements, as several of the genome assemblies were relatively fragmented. RIP was considered present if di-nucleotide frequencies matched the indices: (TpA/ApT) ≥0.89 and (CpA + TpG)/(ApC + GpT) ≤1.03^76^ and visual inspection of RIPCAL alignments showed one or more peaks for (CA ← → TA) + (TG ← → TA) mutations^73^. Additional details about the RIPCAL program can be found in Hane and Oliver^73^.

## Electronic supplementary material

Supplementary Information

Table S4

Table S5

Table S6

Table S7

Table S8

Table S9
