# Supplementary material for: Genome wide analysis of the transition to pathogenic lifestyles in Magnaporthales fungi
Source: Sci Rep. 2018 Apr 12;8:5862. doi: 10.1038/s41598-018-24301-6 (PMC5897359; doi:10.1038/s41598-018-24301-6)
Supplement: Supplementary file 1 — Supplementary Information [file 41598_2018_24301_MOESM1_ESM.pdf]

Genome wide analysis of the transition to pathogenic lifestyles in Magnaporthales fungi

Ning Zhang[1,2*], Guohong Cai[3], Dana C. Price[4], Jo Anne Crouch[5], Pierre Gladieux[6], Bradley Hillman[1], Chang Hyun Khang[7], Marc-Henri LeBrun[8], Yong-Hwan Lee[9], Jing Luo[1], Huan Qiu[10], Daniel Veltri[11], Jennifer H. Wisecaver[12], Jie Zhu[7], Debashish Bhattacharya[2,*]

[1]Department of Plant Biology, Rutgers University, New Brunswick, New Jersey 08901, USA

[2]Department of Biochemistry and Microbiology, Rutgers University, New Brunswick, New Jersey 08901, USA

[3]United States Department of Agriculture, Agricultural Research Service; and Department of Botany and Plant Pathology, Purdue University, West Lafayette, Indiana 47907, USA

[4]Department of Plant Biology, Center for Vector Biology, Rutgers University, New Brunswick, New Jersey 08901, USA

[5]Mycology and Nematology Genetic Diversity and Biology Laboratory, United States Department of Agriculture, Beltsville, Maryland 20705, USA

[6] BGPI, Univ Montpellier, INRA, CIRAD, Montpellier SupAgro, F-34398 Montpellier, France

[7]Department of Plant Biology, University of Georgia, Athens, GA 30602, USA

[8]National Institute of Agricultural Research, Avenue Lucien Brétignières, BP 01, 78850 Thiverval-Grignon, France

[9]Center for Fungal Genetic Resources, Plant Genomics and Breeding Institute, and Research Institute of Agriculture and Life Sciences, Seoul National University, Seoul 08826, Republic of Korea

[10]Department of Ecology, Evolution and Natural Resources, Rutgers University, New Brunswick, New Jersey 08901, USA

[11]Bioinformatics and Computational Biosciences Branch, Office of Cyber Infrastructure and Computational Biology, National Institute of Allergy and Infectious Diseases, NIH, Rockville, MD, USA

[12]Department of Biochemistry, Purdue University, West Lafayette, Indiana, USA

[*]Corresponding authors: Ning Zhang and Debashish Bhattacharya

(e-mails: ningz@rutgers.edu; d.bhattacharya@rutgers.edu)

Supplementary information

Supplementary Information accompanies this paper at online.

Supplementary text

Analysis of TEs in Magnaporthales.

Supplementary figure legends

Supplementary figure S1.

Bacterial-derived HGTs in the Magnaporthales. (A) Phylogenetic tree of a hypothetical protein. (B) Phylogenetic tree of an antibiotic biosynthesis monooxygenase. Statistical support (>0.8) is indicated for each branch. Magnaporthales are shown in brown text and prokaryotes in black text. The clade identity of each major Magnaporthales lineage is shown.

Supplementary figure S2.

(A) Comparison of the total length of transposable element (TE) hits predicted by the REPET v2.5 pipeline as a percentage of genome size across ten fungi in the Magnaporthaceae. The ten fungi are organized according to phylogenetic relatedness, with the most basal species given on the right and most derived species on the left. TEs predicted as Class I are shown in red, Class II in green, and those of unknown class in blue. The proportion of TE hits (Total %/Class I %/Class II %/Unknown %) are as follows: *Pseudohalonectria lignicola* (3.44/2.98/0.45/0.01), *Ophioceras dolichostomum* (1.95/1.34/0.59/0.02), *Magnaporthe oryzae* 70-15 (11.88/7.64/3.71/0.53), *Magnaporthe grisea* (1.45/1.13/0.32/0.00), *Magnaporthe salvinii* M69 (2.07/1.89/0.15/0.04), *Falciphora oryzae* (15.35/11.30/3.67/0.38), *Gaeumannomyces graminis* (6.88/5.67/0.95/0.27), *Magnaporthiopsis incrustans* (4.58/3.90/0.67/0.00), *Magnaporthiopsis poae* (0.64/0.49/0.13/0.03), and *Magnaporthiopsis rhizophila* (8.01/7.62/0.39/0.00).

(B) Comparison of lengths for predicted transposable elements (TEs) grouped by orders as a percentage of genome size for each of the ten Magnaportheaceae genomes. Shown are the Class I orders: LTR, LINE and DIRS, as well as, Class II orders: TIR and Helitron. The “OTHER”

category encompasses all other TEs predicted by REPET for a given genome, including those of unknown class.

Supplementary figure S3.
Comparison of di-nucleotide RIP indices produced by RIPCAL for six TE superfamilies across ten Magnaportheaceae genomes. TE superfamilies based on the Wicker system (Wicker et al. 2007) are arranged in rows by class and order: DIRS (Class I, DIRS-like), I (Class I, LINE), Copia (Class I, LTR), Gypsy (Class I, LTR), Helitron (Class II, Helitron-like), and Tc1-Mariner (Class II, TIR). Evidence for RIP (indicated by *) is supported with index values (TpA / ApT) ≥ 0.89 (red bars) and (CpA + TpG) / (ApC + GpT) ≤ 1.03 (blue bars). These cutoff values are based on previous work on *Neurospora crassa*[76]. Entries left blank either have less than ten respective TE sequences ≥ 80bp or no single sequence ≥ 300 bp as described in Methods. For the results shown, all include one or more sequence over 500 bp in length.

Supplementary table S1.
Raw sequence reads of five species in Magnaporthales.

Supplementary table S2.
Genome assembly and annotation statistics of five species in Magnaporthales.

Supplementary table S3.
CEGMA analysis to identify 248 conserved core eukaryotic genes in assembled genomes of five species in Magnaporthales.

Supplementary table S4.
The 321 cases of HGT that are either supportive or inconclusive (regardless of branch support) that need further investigation.

Supplementary table S5.
Ortholog groups (OGs) that show evidence of positive selection (FDR ≤ .01) in the wood, blast, and root infecting fungal clades.

Supplementary table S6.

Over-represented GO terms shared between the root and blast pathogenic fungal clades that may comprise common gene families that elucidate pathogen adaptation.

Supplementary table S7.

Blast2GO enrichment analyses that highlight 54, 42, and 25 over-represented "most specific" gene ontology (GO) terms in each major fungal clade, respectively.

Supplementary table S8.

List of identifiers of the secretome and small secreted proteins (SSPs), and species-specific SSPs in Magnaporthales species.

Supplementary table S9.

List of identifiers of the clade-specific secretome and small secreted proteins (SSPs) in Magnaporthales species.

Supplementary Text

Transposon Analyses

Analysis of *de novo* TEs: REPET *de novo* predicted TEs from the *TEdenovo* pipeline were clustered using the CD-HIT server to check for similarities across the ten genomes. 580 initial sequences were clustered into 340 clusters containing, at most, 18 sequences with ≥ 80% shared sequence identity. The majority of TE clusters (337) contain TEs from only a single Magnaporthales taxon, suggesting that most of these elements are adapted to their genome of origin, and/or originated after diversification and speciation. Eight TE clusters were identified as containing TEs from two taxa, while one example was found to contain TEs from three and four taxa, respectively (Clusters 21 and 35). BLAST2GO was run on the representative sequence identified by CD-HIT for each cluster using the NCBI *nt* database. Cluster #21, with TEs from four taxa (*G. graminis, M. incrustans, M. poae*, and *M. rhizophila*), was checked using a large retrotransposon derivative (LARD) representative sequence from *M. incrustans* and produced a consensus hit of "proline permease mRNA 13508" (NCBI: XM_009228526.1) from the *G. graminis tritici R3-111a-1* genome. Cluster #35 with TEs from three taxa (*F. oryzae, M. poae*, and *M. rhizophila*) was most similar to an *F. oryzae* unclassified TIR and returned a "telomere partial sequence" result from the *M. oryzae 70-15* genome. Across all clusters, avirulence (AVR) genes from *M. oryzae* were the most common identification (33 hits), including: Pita-1 (19 hits), Pita-2 (2 hits) and Pia (12 hits). This is unsurprising given that AVR genes have been shown in

previous work to occur near TE regions in *M. oryzae* (Singh et al. 2014, Zhang et al. 2014, Chuma et al. 2011).

RIP analysis: Repeat families were assessed for evidence of RIP (Selker 1990; Cambareri et al. 1989) using the RIPCAL program as indicated in Fig. S3 for the DIRS, I, Copia, Gypsy, Helitron and Tc1-Mariner TE superfamilies. With the exception of *O. dolichostomum* and *M. rhizophila,* evidence for RIP was found in at least one TE superfamily: *P. lignicola* (DIRS, Helitron), *M. oryzae* (DIRS, I, Copia, Helitron, Tc1-Mariner)*, M. grisea* (I, Gypsy, Tc1-Mariner)*, M. salvinii* (DIRS, Gypsy)*, F. oryzae* (DIRS, Helitron)*, G. graminis* (Helitron)*, M. incrustans* (DIRS, Helitron)*,* and *M. poae* (Copia, Gypsy). Fig. S3 also shows the di-nucleotide RIP indices (TpA / ApT) and (CpA + TpG) / (ApC + GpT) produced by RIPCAL for the above TE superfamilies. We note cutoffs for the RIP indices used originate from work on *Neurospora crassa,* so it is possible the given taxa might be better characterized using different values.

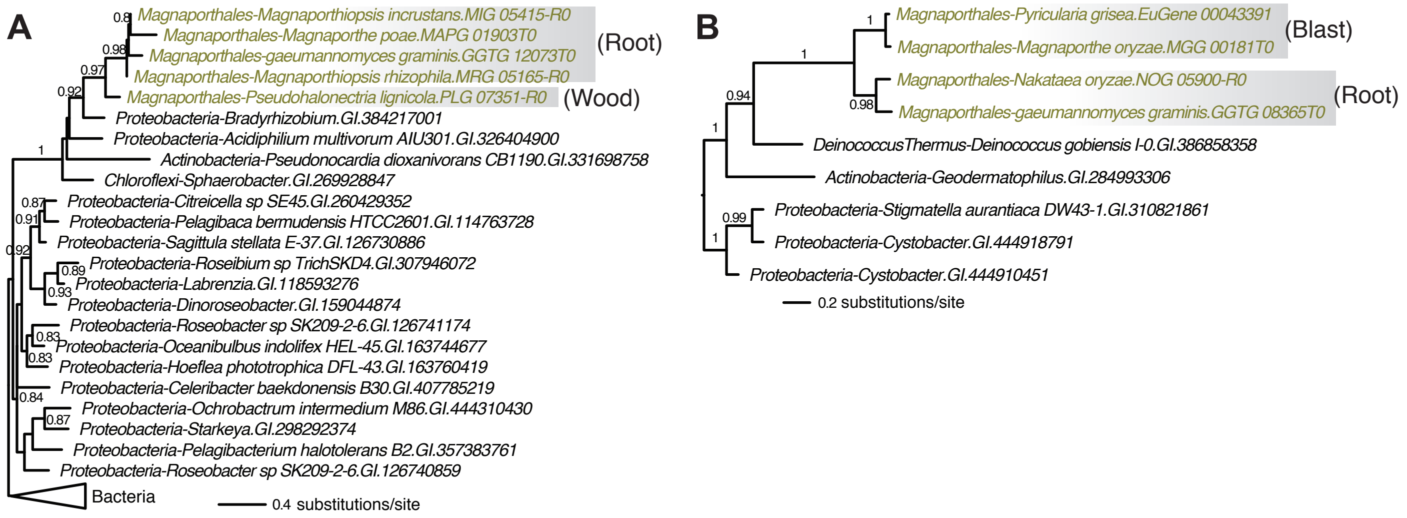
A
Magnaporthales-Magnaporthiopsis incrustans.MIG 05415-R0
Magnaporthales-Magnaporthe poae.MAPG 01903T0
Magnaporthales-gaeumannomyces graminis.GGTG 12073T0
Magnaporthales-Magnaporthiopsis rhizophila.MRG 05165-R0
(Root)
Magnaporthales-Pseudohalonectria lignicola.PLG 07351-R0
(Wood)
Proteobacteria-Bradyrhizobium.GI.384217001
Proteobacteria-Acidiphilium multivorum AIU301.GI.326404900
Actinobacteria-Pseudonocardia dioxanivorans CB1190.GI.331698758
Chloroflexi-Sphaerobacter.GI.269928847
Proteobacteria-Citreicella sp SE45.GI.260429352
Proteobacteria-Pelagibaca bermudensis HTCC2601.GI.114763728
Proteobacteria-Sagittula stellata E-37.GI.126730886
Proteobacteria-Roseibium sp TrichSKD4.GI.307946072
Proteobacteria-Labrenzia.GI.118593276
Proteobacteria-Dinoroseobacter.GI.159044874
Proteobacteria-Roseobacter sp SK209-2-6.GI.126741174
Proteobacteria-Oceanibulbus indolifex HEL-45.GI.163744677
Proteobacteria-Hoeflea phototrophica DFL-43.GI.163760419
Proteobacteria-Celeribacter baekdonensis B30.GI.407785219
Proteobacteria-Ochrobactrum intermedium M86.GI.444310430
Proteobacteria-Starkeya.GI.298292374
Proteobacteria-Pelagibacterium halotolerans B2.GI.357383761
Proteobacteria-Roseobacter sp SK209-2-6.GI.126740859
Bacteria
0.4 substitutions/site
B
Magnaporthales-Pyricularia grisea.EuGene 00043391
Magnaporthales-Magnaporthe oryzae.MGG 00181T0
(Blast)
Magnaporthales-Nakataea oryzae.NOG 05900-R0
Magnaporthales-gaeumannomyces graminis.GGTG 08365T0
(Root)
DeinococcusThermus-Deinococcus gobiensis I-0.GI.386858358
Actinobacteria-Geodermatophilus.GI.284993306
Proteobacteria-Stigmatella aurantiaca DW43-1.GI.310821861
Proteobacteria-Cystobacter.GI.444918791
Proteobacteria-Cystobacter.GI.444910451
0.2 substitutions/site

Fig. S1

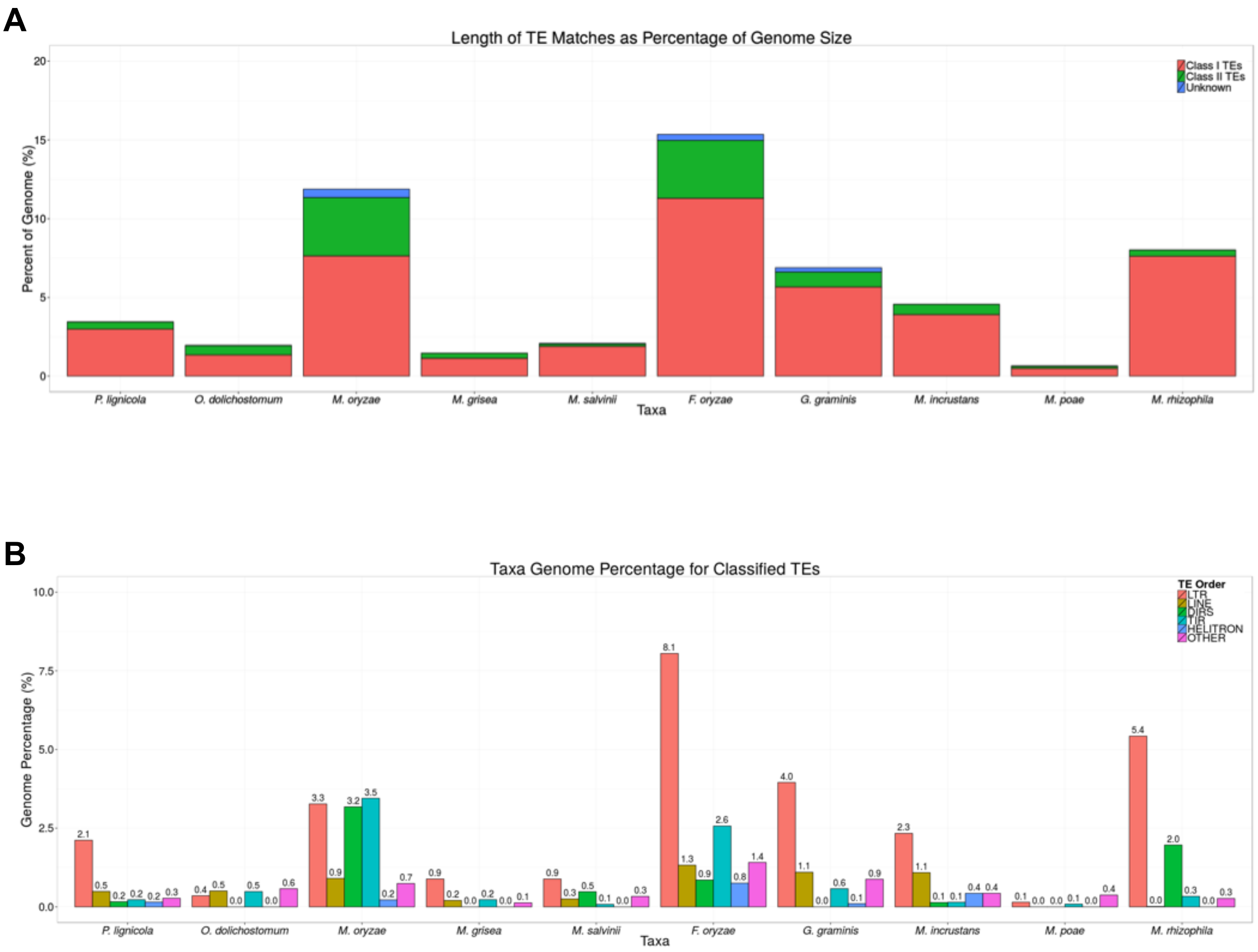
A
Length of TE Matches as Percentage of Genome Size
Class I TEs
Class II TEs
Unknown
Percent of Genome (%)
Taxa
P. lignicola
O. dolichostomum
M. oryzae
M. grisea
M. salvinii
F. oryzae
G. graminis
M. incrustans
M. poae
M. rhizophila
B
Taxa Genome Percentage for Classified TEs
TE Order
LTR
LINE
DIRS
TIR
HELITRON
OTHER
Genome Percentage (%)
Taxa

Fig. S2

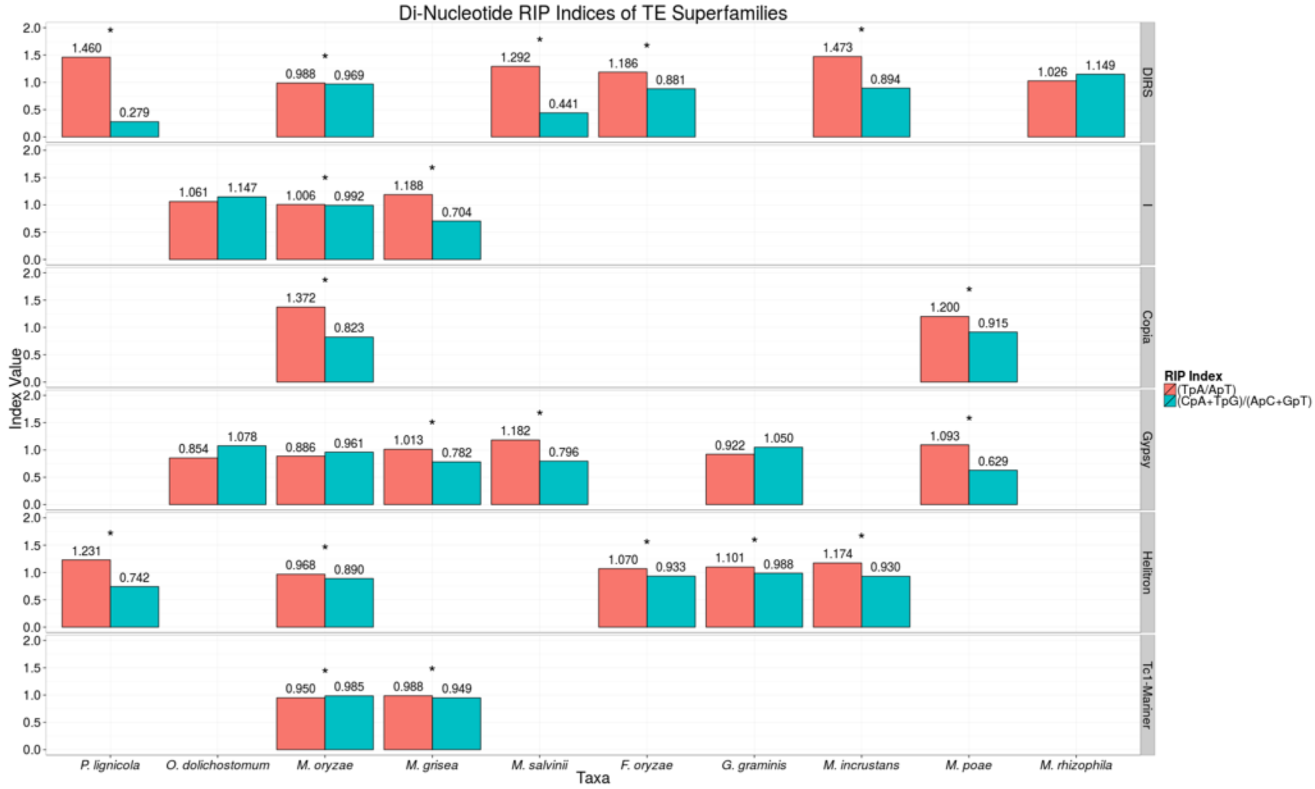

Di-Nucleotide RIP Indices of TE Superfamilies
Index Value
Taxa
DIRS
I
Copia
Gypsy
Helitron
Tc1-Mariner
RIP Index
(TpA/ApT)
(CpA+TpG)/(ApC+GpT)
P. lignicola
O. dolichostomum
M. oryzae
M. grisea
M. salvinii
F. oryzae
G. graminis
M. incrustans
M. poae
M. rhizophila
1.460
0.279
0.988
0.969
1.292
0.441
1.186
0.881
1.473
0.894
1.026
1.149
1.061
1.147
1.006
0.992
1.188
0.704
1.372
0.823
1.200
0.915
0.854
1.078
0.886
0.961
1.013
0.782
1.182
0.796
0.922
1.050
1.093
0.629
1.231
0.742
0.968
0.890
1.070
0.933
1.101
0.988
1.174
0.930
0.950
0.985
0.988
0.949

Fig. S3

Table S1. Raw sequence reads of five species in Magnaporthales.

Species	Strain	Genome sequences		Transcriptome sequences	
		# read pairs	read length	# read pairs	read length
Ophioceras dolichostomum	CBS114926	10,243,331	146	32,612,302	158
Magnaporthiopsis rhizophila	M23	18,996,598	146	28,734,571	158
Magnaporthiopsis incrustans	M35	25,862,061	146	18,109,452	158
Nakataea oryzae	M69	29,578,101	146	16,485,688	158
Pseudohalonectria lignicola	M95	25,160,531	146	17,510,655	158

Table S2. Genome assembly and annotation statistics of five species in Magnaporthales.

Species	Strain	Scaffold		Contig		Assembly Size (Mb)	GC ratio %	# gene models
		N50 (bp)	L50	N50 (bp)	L50			
Ophioceras dolichostomum	CBS114926	97,088	137	47,722	266	43.0	56	12,519
Magnaporthiopsis rhizophila	M23	251,881	52	57,017	268	39.9	54	12,210
Magnaporthiopsis incrustans	M35	164,440	74	59,869	205	39.3	56	12,933
Nakataea oryzae	M69	61,937	179	29,850	344	34.9	58	12,077
Pseudohalonectria lignicola	M95	103,568	122	29,851	188	41.6	54	12,176

Table S3. CEGMA analysis to identify 248 conserved core eukaryotic genes in assembled genomes of five species in Magnaporthales.

Species	Strain	Complete # genes	Complete %	Partial # genes	Partial %	Total %	Missing
Ophioceras dolichostomum	CBS114926	237	0.96	6	0.02	0.98	KOG0276, KOG1185, KOG2311, KOG3232, KOG4392
Magnaporthiopsis rhizophila	M23	235	0.95	6	0.02	0.97	KOG0261, KOG0292, KOG0969, KOG1123, KOG1185, KOG2311, KOG3232
Magnaporthiopsis incrustans	M35	235	0.95	5	0.02	0.97	KOG0261, KOG0291, KOG0434, KOG0969, KOG1123, KOG1185, KOG2311, KOG3232
Nakataea oryzae	M69	236	0.95	5	0.02	0.97	KOG0062, KOG0209, KOG0969, KOG1123, KOG1185, KOG2311, KOG3232
Pseudohalonectria lignicola	M95	236	0.95	6	0.02	0.98	KOG0062, KOG0434, KOG1185, KOG1466, KOG2036, KOG2311
